# Ankyrins and Spectrins in Cardiovascular Biology and Disease

**DOI:** 10.3389/fphys.2017.00852

**Published:** 2017-10-27

**Authors:** Mona M. El Refaey, Peter J. Mohler

**Affiliations:** ^1^Dorothy M. Davis Heart and Lung Research Institute, Wexner Medical Center, The Ohio State University, Columbus, OH, United States; ^2^Department of Physiology & Cell Biology, Wexner Medical Center, The Ohio State University, Columbus, OH, United States; ^3^Department of Internal Medicine, Division of Cardiovascular Medicine, College of Medicine, Wexner Medical Center, The Ohio State University, Columbus, OH, United States

**Keywords:** ankyrin, spectrin, arrhythmia, heart disease, ion channel, signaling

## Abstract

Ankyrins are adaptor proteins critical for the expression and targeting of cardiac membrane proteins, signaling molecules, and cytoskeletal elements. Findings in humans and animal models have highlighted the *in vivo* roles for ankyrins in normal physiology and in cardiovascular disease, most notably in cardiac arrhythmia. For example, human *ANK2* loss-of-function variants are associated with a complex array of electrical and structural phenotypes now termed “ankyrin-B syndrome,” whereas alterations in the ankyrin-G pathway for Na_v_ channel targeting are associated with human Brugada syndrome. Further, both ankyrin-G and -B are now linked with acquired forms of cardiovascular disease including myocardial infarction and atrial fibrillation. Spectrins are ankyrin-associated proteins and recent studies support the critical role of ankyrin-spectrin interactions in normal cardiac physiology as well as regulation of key ion channel and signaling complexes. This review will highlight the roles of ankyrins and spectrins in cardiovascular physiology as well as illustrate the link between the dysfunction in ankyrin- and spectrin-based pathways and disease.

## Introduction

Cardiovascular disease is the leading cause of death in the United States (Rosamond et al., 2008). In 2013, the overall prevalence of death attributable to cardiovascular diseases (CVD) was 222.9 per 100,000 Americans. On the basis of 2013 data, more than 2,200 Americans die of CVD each day, representing an average of 1 death every 40 s. Arrhythmia is the direct cause of death in many cases. Nearly 80% of cases of sudden cardiac death are the result of ventricular arrhythmias (Mehra, 2007). Thus, understanding the molecular mechanisms underlying human arrhythmias has been an active area of cardiovascular research (Cunha and Mohler, 2006), with a major focus on ion channels, transporters and receptors that regulate the action potential at baseline and in disease. Ankyrins and spectrins are adaptor proteins now linked with both electrical and structural forms of heart disease. Here, we will review the roles of ankyrin and spectrin polypeptides as critical components of cardiac myocytes using findings from human and animal models.

## Ankyrin genes

Ankyrins are a family of intracellular adaptor proteins that organize and anchor integral membrane protein complexes to the spectrin- and actin-based cytoskeleton (Bennett and Baines, 2001). In vertebrates, three genes (*ANK1, ANK2, ANK3*), encode ankyrin polypeptides. *ANK1* is located on human chromosome 8p11 and encodes ankyrin-R polypeptides (Lambert et al., 1990). *ANK2* is located on human chromosome 4q25-27 and encodes ankyrin-B polypeptides (Otto et al., 1991). *ANK3* is located on human chromosome 10q21 and encodes ankyrin-G polypeptides (Kordeli et al., 1995). Alternative splicing produces a series of ankyrin gene products with unique subcellular distributions and functional properties (Bennett and Baines, 2001; Cunha and Mohler, 2006; Cunha et al., 2008). For example, alternative splicing of *ANK2* results in 440 kDa and 220 kDa ankyrin-B (Kunimoto, 1995; Hashemi et al., 2009). Ankyrins R, B, and G polypeptides have been identified in ventricular myocytes (Li et al., 1993; Mohler et al., 2002, 2004a,b). However, the full extent of alternative splicing, expression, localization, and function is still under investigation.

## Ankyrin domains and binding partners

**Figure 1:**
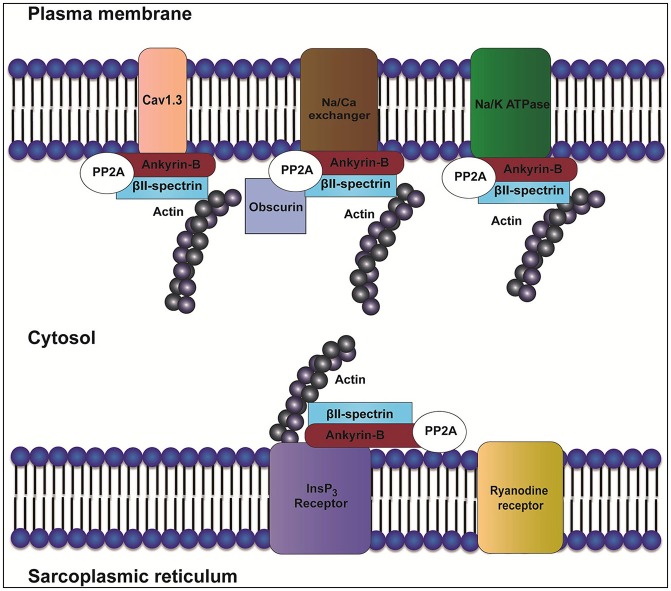
Role of ankyrin-B in localization of the InsP_3_ receptor, Na/K-ATPase, Ca_v_1.3, and NCX. In heart, ankyrin-B targets and localizes ion channels and transporters such as inositol trisphosphate receptor (InsP_3_R), sodium/potassium ATPase (Na/K-ATPase), Ca_v_1.3, and Na/Ca exchanger (NCX). Ankyrin-B also targets protein phosphatase type 2A (PP2A) through its regulatory subunit B56α. Interaction between ankyrin-B and βII spectrin forms a complex that is important for the localization and stability of ion channels and transporters such as Na/K-ATPase, Ca_v_1.3, InsP_3_R, and NCX.

Canonical ankyrins have four domains: a membrane-binding domain (MBD), a spectrin-binding domain (SBD), a death domain (DD) and a C-terminal domain (CTD) (Mohler, 2006). Together, DD and CTD encompass the “regulatory domain” (RD). Ankyrin-B and ankyrin-G are closely related in amino acid sequence with 67% amino acid identity between the MBD, SBD, and DD. Despite this homology and shared expression in cardiac myocytes, ankyrin-B and ankyrin-G maintain differential distributions and non-overlapping functions. For example, while ankyrin-B is required for the localization of the Na/K-ATPase and Na/Ca exchanger (NCX) (Figure 1) to transverse-tubule membranes, ankyrin-G is required for targeting Na_v_1.5 to the intercalated disc (Mohler et al., 2004a; Makara et al., 2014; Wu et al., 2015).

**Figure 2:**
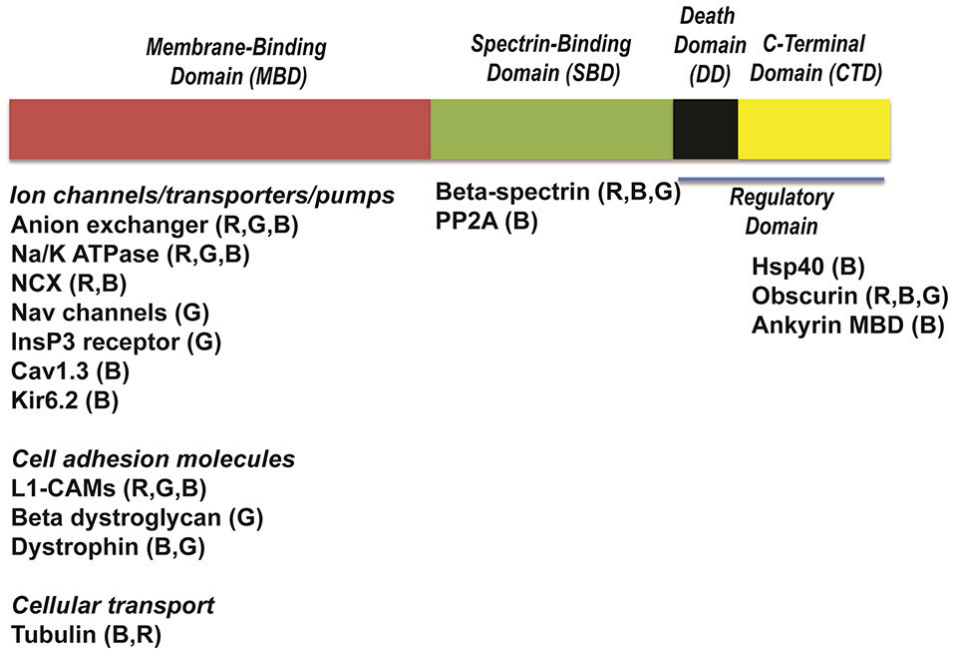
Structure of ankyrins and ankyrin-binding partners. Ankyrins are formed of four distinct domains: a membrane-binding domain (MBD), a spectrin-binding domain (SBD), a death domain (DD) and a C-terminal domain (CTD). Each domain interacts with distinct ion channels, transporters and pumps. Ca_v_1.3, calcium channel, voltage-dependent, L type, alpha 1D subunit; Na/K-ATPase, sodium/potassium ATPase; NCX, Na/Ca exchanger; InsP_3_R, Inositol trisphosphate receptor; Kir6.2, Inward-rectifier potassium ion channel; Na_v_ channels, voltage-gated sodium channels; L1-CAMs, The L1 family of neural cell adhesion molecules; PP2A, protein phosphatase type 2A; Hsp40, Heat shock protein 40.

The MBD is comprised of 24 consecutive *ANK* repeats. In heart, this domain is essential for the interaction with ion channels and transporters (Figure 2) including the Na/K-ATPase (Koob et al., 1988), NCX (Li et al., 1993), voltage-gated Na_v_ channel (Mohler et al., 2004a), the inward rectifier subunit (Kir6.2) (Li et al., 2010), voltage-gated Ca^2+^ channels (Ca_v_1.3) (Cunha et al., 2011), and inositol trisphosphate receptor (InsP_3_ receptor) (Bourguignon et al., 1993; Hortsch et al., 2009). MBD binds to a variety of cell adhesion molecules including members of the L1 family (Davis et al., 1993). The impact of these interactions in the heart is not well studied, but an important area for future research.

The SBD associates with high affinity to β spectrin polypeptides (Bennett and Stenbuck, 1979) via the N-terminal ZU5 (Zu5N) domain (Ipsaro and Mondragon, 2010). This interaction is critical for a number of physiological functions including maintenance of normal erythrocyte cell membrane stability (Bodine et al., 1984). As described later, human *ANK2* variants that alter spectrin-binding are now linked with potentially fatal forms of cardiac arrhythmia (Smith et al., 2015). The SBD also interacts with signaling molecules such as B56α, the regulatory subunit of protein phosphatase 2A (PP2A) (Bhasin et al., 2007; Little et al., 2015).

The ankyrin regulatory domain (RD) interacts with proteins including obscurin. Obscurin is a giant scaffolding protein with key roles in muscle myosin incorporation, A-band formation and lateral alignment of M-lines in adjacent myofibrils (Benian et al., 1996; Borisov et al., 2006; Cunha and Mohler, 2008). Cunha and colleagues demonstrated that ankyrin-B/obscurin interaction recruits PP2A to the cardiac M-line (Cunha and Mohler, 2008) (Figure 1). In addition to the intermolecular interactions, the regulatory domain regulates the intramolecular interactions with the ankyrin MBD (Abdi et al., 2006).

## Spectrin polypeptides in cardiac physiology

Spectrins are actin-associated proteins that provide structural support to the plasma membrane and play roles in the localization of membrane and signaling proteins in the heart (Hund and Mohler, 2010). Spectrin alpha (α) and beta (β) subunits are assembled laterally to form anti-parallel heterodimers, and these heterodimers are assembled to form a flexible hetero-tetramic molecule (Bennett and Baines, 2001). There are two α spectrin genes and five β spectrin genes that encode a diverse group of alternatively-spliced spectrin isoforms with specific functions (Bennett and Healy, 2009). In ventricular myocytes, αII and βII spectrin are found at transverse-tubule and sarcoplasmic reticulum (SR) membranes (Baines and Pinder, 2005), whereas βIV spectrin is located at the intercalated disc (Hund et al., 2010).

In heart, βII spectrin directly interacts with ankyrin-B at transverse-tubule membranes (Mohler et al., 2004c; Smith et al., 2015). In fact, βII spectrin is a critical element of the larger ankyrin-B complex that directly links membrane and signaling proteins including NCX, Na/K ATPase, and β56α with the underlying actin-based cytoskeleton. Loss of βII spectrin in adult ventricular myocytes directly impacts the expression and localization of ankyrin-B as well as ankyrin-B-associated proteins (Smith et al., 2015). Beyond loss of ankyrin-binding proteins, βII spectrin null myocytes unexpectedly showed abnormal localization of the cardiac ryanodine receptor. The relationship of βII spectrin and ryanodine receptor is currently unknown. At the animal level, loss of βII spectrin in mice is lethal in mid-gestation due to incomplete development of the heart (Tang et al., 2003). Conditional deletion of βII spectrin in ventricular myocytes results in sinus node dysfunction and ventricular arrhythmia. These mice also display cytoskeletal remodeling and accelerated heart failure following transverse aortic constriction. As described later, human *ANK2* variants that alter the interaction with βII spectrin are linked with congenital arrhythmia (Smith et al., 2015). Further, βII spectrin levels are altered in acquired forms of human heart failure and arrhythmia, as well as in animal models of cardiovascular disease (Smith et al., 2016). Finally, individuals with Beckwith-Wiedemann syndrome (BWS) with a reduction in βII spectrin expression (Yao et al., 2010) show congenital heart failure and ventricular septal defects (Drut et al., 2006).

In heart, βIV spectrin directly associates with ankyrin-G and is required for the targeting of calcium/calmodulin-dependent kinase II (CaMKII) to the intercalated disc (Hund et al., 2010). Notably, this interaction is critical for the regulation of I_Na_ by modulating CaMKII-dependent phosphorylation of Na_v_1.5 (Hund et al., 2010; Koval et al., 2012). Beyond, ankyrin-G and CaMKII, βIV spectrin regulates TREK-1, a mechano-sensitive K_2P_ channel (Heurteaux et al., 2004). More specifically, βIV spectrin and TREK-1 associate, and βIV spectrin is required for TREK-1 localization and function in ventricular myocytes (Hund et al., 2014). Mice lacking TREK-1 binding display delayed action potential repolarization and arrhythmia. These data support a broader role for βIV spectrin in cardiovascular electrophysiology by regulation of TREK-1 membrane localization and function. Like βII spectrin, βIV spectrin levels are altered in human heart failure as well as in animal models of cardiovascular disease (Hund et al., 2014).

Finally, αII spectrin-deficient embryos are embryonic lethal with abnormal cardiac shape, cardiac dilatation and changes in myocardium (Stankewich et al., 2011). Beyond ankyrins, Mena and VASP, important regulators of actin dynamics form a complex with αII spectrin in adult cardiomyocytes (Benz et al., 2013). The *in vivo* role(s) of αII spectrin in adult heart is currently unknown but an important area for future research.

## *In vivo* roles for cardiac ankyrins

### Ankyrin-G is required for targeting Na_v_1.5

In neurons, the link between ankyrin-G and Na_v_ channel targeting is well established (Jenkins and Bennett, 2001; Saifetiarova et al., 2017). In heart, rat ventricular myocytes lacking ankyrin-G display a reduction in Na_v_1.5 expression, localization, and I_Na_ (Lowe et al., 2008). In fact, in myocytes with reduced ankyrin-G expression, Na_v_1.5 localization is limited in the perinuclear region of rat ventricular myocytes. Notably, this role is specific for ankyrin-G vs. ankyrin-B as ankyrin-B-null neonatal cardiomyocytes show no difference in Na_v_1.5 expression or localization. Further, loss of ankyrin-G is specific for I_Na_ vs. other cardiac currents (e.g., I_Ca, L_) (Lowe et al., 2008).

In animals, ankyrin-G expression is required for the targeting of Na_v_1.5 and CaMKII to the intercalated disc (Makara et al., 2014). Na_v_1.5 expression and I_Na_ is reduced in myocytes derived from mice with selective loss of cardiomyocyte ankyrin-G (cKO) (Makara et al., 2014). Further, βIV spectrin is absent in the intercalated disc of myocytes of ankyrin cKO mice. Electrocardiographic studies of these mice show significant reduction in heart rate, impaired atrioventricular conduction, and an increase in PR interval. Moreover, cKO mice display a significant increase in the QRS interval and delayed intraventricular conduction. Administration of flecainide or catecholamines (elevated adrenergic stimulation) induces arrhythmia in these mice.

Beyond animals, work in humans supports the link between ankyrin-G and Na_v_ channel targeting. Priori et al. (2000) identified an individual with Brugada syndrome harboring an *SCN5A* variant in the Na_v_1.5 motif that interacts with ankyrin-G (Mohler et al., 2004a). The group identified the *SCN5A* variant in a 47-year-old female with a history of palpitations and an unexplained syncope at rest. Using *in vitro* binding assays, the p.E1053K variant was found to disrupt the interaction between Na_v_1.5 and ankyrin-G resulting in abnormal targeting in myocytes.

A canine myocardial infarction (MI) model was used to study the role of ankyrin-G in acquired arrhythmic disease. At 5 days, there was a 42% reduction in Na_v_1.5 protein expression and a 50% increase in ankyrin-G expression in the epicardial border zone. These data suggest that ankyrin-G levels increase to regulate Na_v_1.5 recruitment to sarcolemmal membrane (Dun et al., 2013).

### Ankyrin-B

First identified in brain, ankyrin-B has now been directly linked with a host of functions in the heart. Further, ankyrin-B dysfunction is now well documented in multiple forms of congenital and acquired cardiovascular disease. While ankyrin-B was known to directly associate with critical cardiac ion channels and transporters, an *in vivo* role for ankyrin-B in cardiac function was first illustrated using mice heterozygous for an ankyrin-B null mutation. While ankyrin-B null mice die shortly after birth, ankyrin-B^+/−^ mice live to adulthood but display bradycardia, heart rate variability, prolonged QT intervals, and sudden cardiac death in response to exercise combined with catecholaminergic stimulation (Mohler et al., 2003). While adult ventricular myocytes from ankyrin-B^+/−^ mice display normal action potential duration, these myocytes display early and delayed after-depolarizations in response to catecholamines. At the molecular level, ankyrin-B^+/−^ ventricular myocytes display reduced expression and abnormal localization of ankyrin-B-binding partners including Na/K ATPase and NCX. As illustrated in computational models (Wolf et al., 2010), loss of Na/K ATPase and NCX results in increased SR calcium stores that result in an arrhythmogenic substrate in the ankyrin-B^+/−^ animals (Mohler et al., 2004b). Subsequent work from Despa and colleagues revealed significant alterations in local [Ca^2+^] as well as hyper-phosphorylation of ryanodine receptors in ankyrin-B^+/−^ ventricular myocytes (Camors et al., 2012; Popescu et al., 2016) potentially due to altered PP2A regulation (DeGrande et al., 2012). Further characterization of ankyrin-B^+/−^ mice showed premature senescence and reduced longevity with mice showing loss of adiposity starting at 6 months of age (Mohler et al., 2007a).

Ankyrin-B dysfunction is directly linked with human cardiovascular disease. Over two decades ago, Schott and colleagues identified type 4 long QT syndrome in a large French kindred with sinus node dysfunction and episodes of atrial fibrillation (Schott et al., 1995). Prolonged QTc was observed among the 25 affected patients (21 adults and 4 children). Individuals in the family also displayed catecholaminergic polymorphic ventricular arrhythmia and sudden death. Sequencing of the gene encoding ankyrin-B *(ANK2)* revealed a positional transition (A to G) mutation at position 4,274 in exon 36, leading to the substitution of a glycine for a glutamic acid at amino acid residue 1,425 (p.E1425G) near the regulatory domain of ankyrin-B in forty-five family members (24 carriers and 21 non-carriers). The p.E1425G variant, subsequently identified as a loss-of-function variant in myocytes, showed prevalence in 22 out of 24 individuals with the LQT4 phenotype and was absent in non-affected individuals (Mohler et al., 2003). Since this initial discovery, a number of new ankyrin-B loss-of-function variants have been identified in individuals with ventricular arrhythmias. However, as discussed below, due to the diversity of non-ventricular phenotypes associated with the disease (including many lacking QTc prolongation), the term “ankyrin-B syndrome” has been adopted to describe individuals harboring *ANK2* loss-of-function variants (Mohler et al., 2004b, 2007b). As in the case of many cardiovascular disease genes, the penetrance of phenotypes in *ANK2* variant carriers is variable. As ankyrin-B polypeptide stability and function is directly impacted by physiological and pathological stress (e.g., inflammation, elevated reactive oxygen species), we predict that a “second hit” may be necessary for full disease penetrance.

Active areas of research in this field are currently focused on identifying the mechanisms underlying both congenital and acquired ankyrin-B loss in human disease as well as the discovery of new variants. For example, while loss-of-function variants were limited to the RD, new variants in the ankyrin-B MBD have shed light on new phenotypes associated with “ankyrin-B syndrome.” Recently, a novel MBD variant in the *ANK2* gene (p.S646F) was identified in two Gitxan families with LQTS phenotypes by the Swayne and Arbour groups (Swayne et al., 2017). The first person identified was a 28-year old man with a clinical diagnosis of LQTS and recurrent syncope. Five of this patient's relatives were confirmed to harbor this variant. This variant was also identified in a second family with a clinical diagnosis of LQTS. Within these two families, a total of 13 adults and 5 children have been identified with this novel *ANK2* variant. *In vitro* evaluation illustrated that the expression of ankyrin MBD with p.S646F variant in bacteria was normal and MBD structural properties were intact. However, expression of ankyrin-B p.S646F variant in neonatal cardiomyocytes showed a reduction in ankyrin-B expression and alteration of expression of NCX (Swayne et al., 2017). Finally, beyond LQT and catecholaminergic polymorphic ventricular tachycardia, *ANK2* variants have been associated with other forms of ventricular arrhythmia including early repolarization syndrome (Krogh Broendberg et al., 2015).

*ANK2* variants are not limited to MBD or RD. Smith et al. identified an *ANK2* arrhythmia variant (c.2969G>A) caused by the substitution of Arg to Gln at position 990 (p. R990Q). This variant is rare with a minor allele frequency of 0.007% and resides near the central ZU5 binding surface for βII spectrin. Using myocytes from ankyrin-B conditional knockout mice (cKO), exogenous expression of WT ankyrin-B, but not ankyrin-B p.R990Q was able to rescue the localization of NCX (Smith et al., 2015).

Ankyrin-B dysfunction is also linked with non-ventricular forms of human arrhythmia. Individuals with select *ANK2* loss-of-function variants display atrial fibrillation and/or sinus node dysfunction (Le Scouarnec et al., 2008; Cunha et al., 2011). Consistent with these data, ankyrin-B^+/−^ mice also display both atrial and sinus node phenotypes. At the cellular level, primary atrial and sinus node myocytes from ankyrin-B^+/−^ mice display shortened action potentials and loss of L-type Ca^2+^ current. In ankyrin-B^+/−^ atrial myocytes, voltage-gated L-type Ca^2+^ channel Ca_v_1.3 (but not Ca_v_1.2) expression and function were reduced.

Until recently, *ANK2*-based disease was linked only to individuals with single “point mutations” (Mohler et al., 2004b, 2007b; Sherman et al., 2005; Krogh Broendberg et al., 2015; Swayne et al., 2017). However, recently a family was identified with an *ANK2* transection in chromosome 4 (Huq et al., 2017). The proband had a balanced translocation between the long arms of chromosomes 4 and 9. Five other family members were carriers of the same balanced translocation. The 16-week-old fetus of the proband from a terminated pregnancy was found to have multiple cardiac abnormalities. The proband showed normal echocardiography findings and only Holter monitor recorded few episodes of bradycardia at night. Isolated lymphoblasts from the proband demonstrated a significant reduction in ankyrin-B and binding partners, confirming a model of ankyrin-B haploinsufficiency. The mother of the proband was also a carrier of the translocation and displayed frequent ventricular ectopy and periods of bigeminy.

Beyond congenital disease, dysfunction in ankyrin-B-based pathways has been linked with acquired forms of cardiovascular disease. In a canine model of myocardial infarction, ankyrin-B mRNA levels were increased and protein levels were decreased in day 5-post infarction in the border zone (Hund et al., 2009). At the myocyte level, abnormal distribution of ankyrin-B was noted. The authors further showed changes in expression and/or distribution of downstream ankyrin-associated proteins Na/K ATPase, NCX, InsP_3_ receptor and PP2A (Hund et al., 2009). In addition to its role in myocardial infarction, Li et al. identified the association of ATP-sensitive K^+^ channel (K_ATP_) with ankyrin-B as a cardio-protective mechanism against acute ischemia (Li et al., 2010). Ankyrin-B levels are now also known to be reduced in patients with atrial fibrillation (Cunha et al., 2011).

### Ankyrin-R

To date, human variants in *ANK1* have not been directly linked with cardiac arrhythmia phenotypes. While not the focus on this review, it is of note that variants in *ANK1* are a primary cause for hereditary spherocytosis (Eber et al., 1996; Tse and Lux, 1999; Perrotta et al., 2008; Satchwell et al., 2016).

## Summary

Cardiac ankyrins and spectrins are key multifunctional molecules in the heart. In myocytes, ankyrins, and spectrins serve essential roles in targeting ion channels and transporters to critical membrane domains. However, beyond membrane proteins, these molecules serve as crucial cellular nodes to integrate membrane proteins with critical signaling and structural proteins in diverse cardiac cell types. While animals have served as important models to define ankyrin and spectrin function *in vivo*, more recent findings in patients have both solidified the importance of ankyrins and spectrin in human physiology and disease, and raised important questions related to unexpected roles of these polypeptides. Key future questions in the field remain the impact of environment or “second hit” gene variants on disease susceptibility/penetrance in *ANK2* variant carriers, as well as potential non-myocyte/non-cardiac phenotypes in patients with ankyrin gene variants.

## Author contributions

All authors listed, have made substantial, direct and intellectual contribution to the work, and approved it for publication.

### Conflict of interest statement

The authors declares that the research was conducted in the absence of any commercial or financial relationships that could be construed as a potential conflict of interest.

## Abbreviations

MBD: Membrane-binding domain
CTD: C-terminal domain
SBD: Spectrin-binding domain
DD: Death domain
LQTS: Long QT syndrome
NCX: Sodium-Calcium exchanger
Na/K ATPase: Sodium-potassium ATPase
IP3R: Inositol trisphosphate receptor
cKO: Conditional knock-out
INa,L: Late component of Nav current
CaMKII: Calcium/calmodulin-dependent kinase
KATP: ATP-sensitive K+ channel
Cav1.3: Voltage gated L-type Ca2+ channel
PP2A: Protein phosphatase 2A
Nav1.5: Voltage-gated sodium channel
TREK-1: TWIK-related potassium channel-1.

## References

[B1] Abdi K. M. Mohler P. J. Davis J. Q. Bennett V. (2006). Isoform specificity of ankyrin-B: a site in the divergent C-terminal domain is required for intramolecular association. J. Biol. Chem. 281, 5741–5749. 10.1074/jbc.M506697200 16368689

[B2] Baines A. J. Pinder J. C. (2005). The spectrin-associated cytoskeleton in mammalian heart. Front. Biosci. 10, 3020–3033. 10.2741/1759 15970557

[B3] Benian G. M. Tinley T. L. Tang X. Borodovsky M. (1996). The Caenorhabditis elegans gene unc-89, required fpr muscle M-line assembly, encodes a giant modular protein composed of Ig and signal transduction domains. J. Cell Biol. 132, 835–848. 10.1083/jcb.132.5.835 8603916 PMC2120741

[B4] Bennett V. Baines A. J. (2001). Spectrin and ankyrin-based pathways: metazoan inventions for integrating cells into tissues. Physiol. Rev. 81, 1353–1392. Available online at: http://physrev.physiology.org/content/81/3/1353.full-text.pdf+html 11427698 10.1152/physrev.2001.81.3.1353

[B5] Bennett V. Healy J. (2009). Membrane domains based on ankyrin and spectrin associated with cell-cell interactions. Cold Spring Harb. Perspect. Biol. 1:a003012. 10.1101/cshperspect.a003012 20457566 PMC2882121

[B6] Bennett V. Stenbuck P. J. (1979). The membrane attachment protein for spectrin is associated with band 3 in human erythrocyte membranes. Nature 280, 468–473. 10.1038/280468a0 379653

[B7] Benz P. M. Merkel C. J. Offner K. Abeßer M. Ullrich M. Fischer T.. (2013). Mena/VASP and alphaII-spectrin complexes regulate cytoplasmic actin networks in cardiomyocytes and protect from conduction abnormalities and dilated cardiomyopathy. Cell Commun. Signal 11:56. 10.1186/1478-811X-11-56 23937664 PMC3751641

[B8] Bhasin N. Cunha S. R. Mudannayake M. Gigena M. S. Rogers T. B. Mohler P. J. (2007). Molecular basis for PP2A regulatory subunit B56alpha targeting in cardiomyocytes. Am. J. Physiol. Heart Circ. Physiol. 293, H109–H119. 10.1152/ajpheart.00059.2007 17416611

[B9] Bodine D. M. Birkenmeier C. S. Barker J. E. (1984). Spectrin deficient inherited hemolytic anemias in the mouse: characterization by spectrin synthesis and mRNA activity in reticulocytes. Cell 37, 721–729. 10.1016/0092-8674(84)90408-2 6234993

[B10] Borisov A. B. Sutter S. B. Kontrogianni-Konstantopoulos A. Bloch R. J. Westfall M. V. Russell M. W. (2006). Essential role of obscurin in cardiac myofibrillogenesis and hypertrophic response: evidence from small interfering RNA-mediated gene silencing. Histochem. Cell Biol. 125, 227–238. 10.1007/s00418-005-0069-x 16205939

[B11] Bourguignon L. Y. Jin H. Iida N. Brandt N. R. Zhang S. H. (1993). The involvement of ankyrin in the regulation of inositol 1,4,5-trisphosphate receptor-mediated internal Ca2+ release from Ca2+ storage vesicles in mouse T-lymphoma cells. J. Biol. Chem. 268, 7290–7297. 8385102

[B12] Camors E. Mohler P. J. Bers D. M. Despa S. (2012). Ankyrin-B reduction enhances Ca spark-mediated SR Ca release promoting cardiac myocyte arrhythmic activity. J. Mol. Cell Cardiol. 52, 1240–1248. 10.1016/j.yjmcc.2012.02.010 22406428 PMC3348355

[B13] Cunha S. R. Hund T. J. Hashemi S. Voigt N. Li N. Wright P.. (2011). Defects in ankyrin-based membrane protein targeting pathways underlie atrial fibrillation. Circulation 124, 1212–1222. 10.1161/CIRCULATIONAHA.111.023986 21859974 PMC3211046

[B14] Cunha S. R. Le Scouarnec S. Schott J. J. Mohler P. J. (2008). Exon organization and novel alternative splicing of the human ANK2 gene: implications for cardiac function and human cardiac disease. J. Mol. Cell Cardiol. 45, 724–734. 10.1016/j.yjmcc.2008.08.005 18790697 PMC2630508

[B15] Cunha S. R. Mohler P. J. (2006). Cardiac ankyrins: Essential components for development and maintenance of excitable membrane domains in heart. Cardiovasc. Res. 71, 22–29. 10.1016/j.cardiores.2006.03.018 16650839

[B16] Cunha S. R. Mohler P. J. (2008). Obscurin targets ankyrin-B and protein phosphatase 2A to the cardiac M-line. J. Biol. Chem. 283, 31968–31980. 10.1074/jbc.M806050200 18782775 PMC2581558

[B17] Davis J. Q. McLaughlin T. Bennett V. (1993). Ankyrin-binding proteins related to nervous system cell adhesion molecules: candidates to provide transmembrane and intercellular connections in adult brain. J. Cell Biol. 121, 121–133. 10.1083/jcb.121.1.121 8458865 PMC2119766

[B18] DeGrande S. Nixon D. Koval O. Curran J. W. Wright P. Wang Q.. (2012). CaMKII inhibition rescues proarrhythmic phenotypes in the model of human ankyrin-B syndrome. Heart Rhythm 9, 2034–2041. 10.1016/j.hrthm.2012.08.026 23059182 PMC3630478

[B19] Drut R. Quijano G. Altamirano M. E. Jones M. C. Maffessoli O. B. (2006). Vascular malformation and choroid plexus adrenal heterotopia: new findings in Beckwith-Wiedemann syndrome? Fetal Pediatr. Pathol. 25, 191–197. 10.1080/15513810601015704 17162526

[B20] Dun W. Lowe J. S. Wright P. Hund T. J. Mohler P. J. Boyden P. A. (2013). Ankyrin-G participates in INa remodeling in myocytes from the border zones of infarcted canine heart. PLoS ONE 8:e78087. 10.1371/journal.pone.0078087 24155982 PMC3796465

[B21] Eber S. W. Gonzalez J. M. Lux M. L. Scarpa A. L. Tse W. T. Dornwell M.. (1996). Ankyrin-1 mutations are a major cause of dominant and recessive hereditary spherocytosis. Nat. Genet. 13, 214–218. 10.1038/ng0696-214 8640229

[B22] Hashemi S. M. Hund T. J. Mohler P. J. (2009). Cardiac ankyrins in health and disease. J. Mol. Cell Cardiol. 47, 203–209. 10.1016/j.yjmcc.2009.04.010 19394342 PMC2745072

[B23] Heurteaux C. Guy N. Laigle C. Blondeau N. Duprat F. Mazzuca M.. (2004). TREK-1, a K+ channel involved in neuroprotection and general anesthesia. EMBO J. 23, 2684–2695. 10.1038/sj.emboj.7600234 15175651 PMC449762

[B24] Hortsch M. Nagaraj K. Godenschwege T. A. (2009). The interaction between L1-type proteins and ankyrins–a master switch for L1-type CAM function. Cell Mol. Biol. Lett. 14, 57–69. 10.2478/s11658-008-0035-4 18839070 PMC2615246

[B25] Hund T. J. Koval O. M. Li J. Wright P. J. Qian L. Snyder J. S.. (2010). A beta(IV)-spectrin/CaMKII signaling complex is essential for membrane excitability in mice. J. Clin. Invest. 120, 3508–3519. 10.1172/JCI43621 20877009 PMC2947241

[B26] Hund T. J. Mohler P. J. (2010). Cardiac spectrins: alternative splicing encodes functional diversity. J. Mol. Cell Cardiol. 48, 1031–1032. 10.1016/j.yjmcc.2010.02.002 20144617 PMC2866816

[B27] Hund T. J. Snyder J. S. Wu X. Glynn P. Koval O. M. Onal B.. (2014). beta(IV)-Spectrin regulates TREK-1 membrane targeting in the heart. Cardiovasc. Res. 102, 166–175. 10.1093/cvr/cvu008 24445605 PMC3958619

[B28] Hund T. J. Wright P. J. Dun W. Snyder J. S. Boyden P. A. Mohler P. J. (2009). Regulation of the ankyrin-B-based targeting pathway following myocardial infarction. Cardiovasc. Res. 81, 742–749. 10.1093/cvr/cvn348 19074823 PMC2642599

[B29] Huq A. J. Pertile M. D. Davis A. M. Landon H. James P. A. Kline C. F.. (2017). A novel mechanism for human cardiac Ankyrin-B syndrome due to reciprocal chromosomal translocation. Heart Lung Circ. 26, 612–618. 10.1016/j.hlc.2016.09.013 27916589 PMC5413386

[B30] Ipsaro J. J. Mondragón A. (2010). Structural basis for spectrin recognition by ankyrin. Blood 115, 4093–4101. 10.1182/blood-2009-11-255604 20101027 PMC2875089

[B31] Jenkins S. M. Bennett V. (2001). Ankyrin-G coordinates assembly of the spectrin-based membrane skeleton, voltage-gated sodium channels, and L1 CAMs at Purkinje neuron initial segments. J. Cell Biol. 155, 739–746. 10.1083/jcb.200109026 11724816 PMC2150881

[B32] Koob R. Zimmermann M. Schoner W. Drenckhahn D. (1988). Colocalization and coprecipitation of ankyrin and Na+,K+-ATPase in kidney epithelial cells. Eur. J. Cell Biol. 45, 230–237. 2835237

[B33] Kordeli E. Lambert S. Bennett V. (1995). AnkyrinG. a new ankyrin gene with neural-specific isoforms localized at the axonal initial segment and node of ranvier. J. Biol. Chem. 270, 2352–2359. 10.1074/jbc.270.5.2352 7836469

[B34] Koval O. M. Snyder J. S. Wolf R. M. Pavlovicz R. E. Glynn P. Curran J.. (2012). Ca2+/calmodulin-dependent protein kinase II-based regulation of voltage-gated Na+ channel in cardiac disease. Circulation 126, 2084–2094. 10.1161/CIRCULATIONAHA.112.105320 23008441 PMC3811023

[B35] Krogh Broendberg A. Pedersen L. N. Nielsen J. C. Jensen H. K. (2015). Ankyrin-2 variants associated with idiopathic ventricular fibrillation storm in patients with intermittent early repolarization pattern. HeartRhythm Case Rep. 1, 337–341. 10.1016/j.hrcr.2015.05.008 28491579 PMC5419664

[B36] Kunimoto M. (1995). A neuron-specific isoform of brain ankyrin, 440-kD ankyrinB, is targeted to the axons of rat cerebellar neurons. J. Cell Biol. 131(6 Pt 2), 1821–1829. 10.1083/jcb.131.6.1821 8557748 PMC2120681

[B37] Lambert S. Yu H. Prchal J. T. Lawler J. Ruff P. Speicher D.. (1990). cDNA sequence for human erythrocyte ankyrin. Proc. Natl. Acad. Sci. U.S.A. 87, 1730–1734. 10.1073/pnas.87.5.1730 1689849 PMC53556

[B38] Le Scouarnec S. Bhasin N. Vieyres C. Hund T. J. Cunha S. R. Koval O.. (2008). Dysfunction in ankyrin-B-dependent ion channel and transporter targeting causes human sinus node disease. Proc. Natl. Acad. Sci. U. S. A. 105, 15617–15622. 10.1073/pnas.0805500105 18832177 PMC2563133

[B39] Li J. Kline C. F. Hund T. J. Anderson M. E. Mohler P. J. (2010). Ankyrin-B regulates Kir6.2 membrane expression and function in heart. J. Biol. Chem. 285, 28723–28730. 10.1074/jbc.M110.147868 20610380 PMC2937900

[B40] Li Z. P. Burke E. P. Frank J. S. Bennett V. Philipson K. D. (1993). The cardiac Na+-Ca2+ exchanger binds to the cytoskeletal protein ankyrin. J. Biol. Chem. 268, 11489–11491. 8505285

[B41] Little S. C. Curran J. Makara M. A. Kline C. F. Ho H. T. Xu Z.. (2015). Protein phosphatase 2A regulatory subunit B56alpha limits phosphatase activity in the heart. Sci Signal 8, ra72. 10.1126/scisignal.aaa5876 26198358 PMC4680974

[B42] Lowe J. S. Palygin O. Bhasin N. Hund T. J. Boyden P. A. Shibata E.. (2008). Voltage-gated Nav channel targeting in the heart requires an ankyrin-G dependent cellular pathway. J. Cell Biol. 180, 173–186. 10.1083/jcb.200710107 18180363 PMC2213608

[B43] Makara M. A. Curran J. Little S. C. Musa H. Polina I. Smith S. A.. (2014). Ankyrin-G coordinates intercalated disc signaling platform to regulate cardiac excitability *in vivo*. Circ. Res. 115, 929–938. 10.1161/CIRCRESAHA.115.305154 25239140 PMC4224970

[B44] Mehra R. (2007). Global public health problem of sudden cardiac death. J. Electrocardiol. 40, S118–122. 10.1016/j.jelectrocard.2007.06.023 17993308

[B45] Mohler P. J. (2006). Ankyrins and human disease: what the electrophysiologist should know. J. Cardiovasc. Electrophysiol. 17, 1153–1159. 10.1111/j.1540-8167.2006.00540.x 16800854

[B46] Mohler P. J. Gramolini A. O. Bennett V. (2002). The ankyrin-B C-terminal domain determines activity of ankyrin-B/G chimeras in rescue of abnormal inositol 1,4,5-trisphosphate and ryanodine receptor distribution in ankyrin-B (-/-) neonatal cardiomyocytes. J. Biol. Chem. 277, 10599–10607. 10.1074/jbc.M110958200 11781319

[B47] Mohler P. J. Healy J. A. Xue H. Puca A. A. Kline C. F. Allingham R. R.. (2007a). Ankyrin-B syndrome: enhanced cardiac function balanced by risk of cardiac death and premature senescence. PLoS ONE 2:e1051. 10.1371/journal.pone.0001051 17940615 PMC2013943

[B48] Mohler P. J. Le Scouarnec S. Denjoy I. Lowe J. S. Guicheney P. Caron L.. (2007b). Defining the cellular phenotype of “ankyrin-B syndrome” variants: human ANK2 variants associated with clinical phenotypes display a spectrum of activities in cardiomyocytes. Circulation 115, 432–441. 10.1161/CIRCULATIONAHA.106.656512 17242276

[B49] Mohler P. J. Rivolta I. Napolitano C. LeMaillet G. Lambert S. Priori S. G.. (2004a). Nav1.5 E1053K mutation causing Brugada syndrome blocks binding to ankyrin-G and expression of Nav1.5 on the surface of cardiomyocytes. Proc. Natl. Acad. Sci. U.S.A. 101, 17533–17538. 10.1073/pnas.0403711101 15579534 PMC536011

[B50] Mohler P. J. Schott J. J. Gramolini A. O. Dilly K. W. Guatimosim S. duBell W. H.. (2003). Ankyrin-B mutation causes type 4 long-QT cardiac arrhythmia and sudden cardiac death. Nature 421, 634–639. 10.1038/nature01335 12571597

[B51] Mohler P. J. Splawski I. Napolitano C. Bottelli G. Sharpe L. Timothy K.. (2004b). A cardiac arrhythmia syndrome caused by loss of ankyrin-B function. Proc. Natl. Acad. Sci. U.S.A. 101, 9137–9142. 10.1073/pnas.0402546101 15178757 PMC428486

[B52] Mohler P. J. Yoon W. Bennett V. (2004c). Ankyrin-B targets beta2-spectrin to an intracellular compartment in neonatal cardiomyocytes. J. Biol. Chem. 279, 40185–40193. 10.1074/jbc.M406018200 15262991

[B53] Otto E. Kunimoto M. McLaughlin T. Bennett V. (1991). Isolation and characterization of cDNAs encoding human brain ankyrins reveal a family of alternatively spliced genes. J. Cell Biol. 114, 241–253. 10.1083/jcb.114.2.241 1830053 PMC2289074

[B54] Perrotta S. Gallagher P. G. Mohandas N. (2008). Hereditary spherocytosis. Lancet 372, 1411–1426. 10.1016/S0140-6736(08)61588-3 18940465

[B55] Popescu I. Galice S. Mohler P. J. Despa S. (2016). Elevated local [Ca2+] and CaMKII promote spontaneous Ca2+ release in ankyrin-B-deficient hearts. Cardiovasc. Res. 111, 287–294. 10.1093/cvr/cvw093 27131508 PMC4957489

[B56] Priori S. G. Napolitano C. Gasparini M. Pappone C. Della Bella P. Brignole M.. (2000). Clinical and genetic heterogeneity of right bundle branch block and ST-segment elevation syndrome: a prospective evaluation of 52 families. Circulation 102, 2509–2515. 10.1161/01.CIR.102.20.2509 11076825

[B57] Rosamond W. Flegal K. Furie K. Go A. Greenlund K. Haase N.. (2008). Heart disease and stroke statistics−2008 update: a report from the American Heart Association Statistics Committee and Stroke Statistics Subcommittee. Circulation 117, e25–e146. 10.1161/CIRCULATIONAHA.107.187998 18086926

[B58] Saifetiarova J. Taylor A. M. Bhat M. A. (2017). Early and late loss of the Cytoskeletal Scaffolding protein, Ankyrin G reveals its role in maturation and maintenance of nodes of Ranvier in Myelinated Axons. J. Neurosci. 37, 2524–2538. 10.1523/JNEUROSCI.2661-16.2017 28148727 PMC5354314

[B59] Satchwell T. J. Bell A. J. Hawley B. R. Pellegrin S. Mordue K. E. van Deursen C. T.. (2016). Severe Ankyrin-R deficiency results in impaired surface retention and lysosomal degradation of RhAG in human erythroblasts. Haematologica 101, 1018–1027. 10.3324/haematol.2016.146209 27247322 PMC5060018

[B60] Schott J. J. Charpentier F. Peltier S. Foley P. Drouin E. Bouhour J. B.. (1995). Mapping of a gene for long QT syndrome to chromosome 4q25-27. Am. J. Hum. Genet. 57, 1114–1122. 7485162 PMC1801360

[B61] Sherman J. Tester D. J. Ackerman M. J. (2005). Targeted mutational analysis of ankyrin-B in 541 consecutive, unrelated patients referred for long QT syndrome genetic testing and 200 healthy subjects. Heart Rhythm 2, 1218–1223. 10.1016/j.hrthm.2005.07.026 16253912

[B62] Smith S. A. Hughes L. D. Kline C. F. Kempton A. N. Dorn L. E. Curran J.. (2016). Dysfunction of the beta2-spectrin-based pathway in human heart failure. Am. J. Physiol. Heart Circ. Physiol. 310, H1583–H1591. 10.1152/ajpheart.00875.2015 27106045 PMC4935521

[B63] Smith S. A. Sturm A. C. Curran J. Kline C. F. Little S. C. Bonilla I. M.. (2015). Dysfunction in the betaII spectrin-dependent cytoskeleton underlies human arrhythmia. Circulation 131, 695–708. 10.1161/CIRCULATIONAHA.114.013708 25632041 PMC4342332

[B64] Stankewich M. C. Cianci C. D. Stabach P. R. Ji L. Nath A. Morrow J. S. (2011). Cell organization, growth, and neural and cardiac development require alphaII-spectrin. J. Cell Sci. 124(Pt 23), 3956–3966. 10.1242/jcs.080374 22159418 PMC3244980

[B65] Swayne L. A. Murphy N. P. Asuri S. Chen L. Xu X. McIntosh S.. (2017). Novel variant in the ANK2 membrane-binding domain is associated with Ankyrin-B syndrome and structural heart disease in a first nations population with a high rate of long QT syndrome. Circ. Cardiovasc. Genet. 10:e001537. 10.1161/CIRCGENETICS.116.001537 28196901 PMC5312931

[B66] Tang Y. Katuri V. Dillner A. Mishra B. Deng C. X. Mishra L. (2003). Disruption of transforming growth factor-beta signaling in ELF beta-spectrin-deficient mice. Science 299, 574–577. 10.1126/science.1075994 12543979

[B67] Tse W. T. Lux S. E. (1999). Red blood cell membrane disorders. Br. J. Haematol. 104, 2–13. 10.1111/j.1365-2141.1999.01130.x 10027705

[B68] Wolf R. M. Mitchell C. C. Christensen M. D. Mohler P. J. Hund T. J. (2010). Defining new insight into atypical arrhythmia: a computational model of ankyrin-B syndrome. Am. J. Physiol. Heart Circ. Physiol. 299, H1505–H1514. 10.1152/ajpheart.00503.2010 20729400 PMC2993217

[B69] Wu H. C. Yamankurt G. Luo J. Subramaniam J. Hashmi S. S. Hu H.. (2015). Identification and characterization of two ankyrin-B isoforms in mammalian heart. Cardiovasc. Res. 107, 466–477. 10.1093/cvr/cvv184 26109584 PMC4540146

[B70] Yao Z. X. Jogunoori W. Choufani S. Rashid A. Blake T. Yao W.. (2010). Epigenetic silencing of beta-spectrin, a TGF-beta signaling/scaffolding protein in a human cancer stem cell disorder: Beckwith-Wiedemann syndrome. J. Biol. Chem. 285, 36112–36120. 10.1074/jbc.M110.162347 20739274 PMC2975233

